# Isoflurane Exposure Induces Cell Death, Microglial Activation and Modifies the Expression of Genes Supporting Neurodevelopment and Cognitive Function in the Male Newborn Piglet Brain

**DOI:** 10.1371/journal.pone.0166784

**Published:** 2016-11-29

**Authors:** Kevin D. Broad, Jane Hassell, Bobbi Fleiss, Go Kawano, Mojgan Ezzati, Eridan Rocha-Ferreira, Mariya Hristova, Kate Bennett, Igor Fierens, Ryan Burnett, Badr Chaban, Daniel Alonso-Alconada, Aaron Oliver-Taylor, Ilias Tachsidis, Jamshid Rostami, Pierre Gressens, Robert D. Sanders, Nicola J. Robertson

**Affiliations:** 1 Institute for Women’s Health, University College London, London, United Kingdom; 2 Centre for the Developing Brain, Kings College, St Thomas’s Campus, London, United Kingdom; 3 Inserm, Paris, France; 4 University Paris Diderot, Sorbonne Paris Cite, Paris, France; 5 Department of Medical Physics and Biomedical Engineering, University College London, London, United Kingdom; 6 Department of Anesthesiology, University of Wisconsin, Madison, United States of America; 7 Wellcome Department of Imaging Neuroscience, University College London, London, United Kingdom; 8 Surgical Outcomes Research Centre, University College London Hospital, London, United Kingdom; Imperial College London, UNITED KINGDOM

## Abstract

Exposure of the brain to general anesthesia during early infancy may adversely affect its neural and cognitive development. The mechanisms mediating this are complex, incompletely understood and may be sexually dimorphic, but include developmentally inappropriate apoptosis, inflammation and a disruption to cognitively salient gene expression. We investigated the effects of a 6h isoflurane exposure on cell death, microglial activation and gene expression in the male neonatal piglet brain. Piglets (n = 6) were randomised to: (i) naive controls or (ii) 6h isoflurane. Cell death (TUNEL and caspase-3) and microglial activation were recorded in 7 brain regions. Changes in gene expression (microarray and qPCR) were assessed in the cingulate cortex. Electroencephalography (EEG) was recorded throughout. Isoflurane anesthesia induced significant increases in cell death in the cingulate and insular cortices, caudate nucleus, thalamus, putamen, internal capsule, periventricular white matter and hippocampus. Dying cells included both neurons and oligodendrocytes. Significantly, microglial activation was observed in the insula, pyriform, hippocampus, internal capsule, caudate and thalamus. Isoflurane induced significant disruption to the expression of 79 gene transcripts, of these 26 are important for the control of transcription and 23 are important for the mediation of neural plasticity, memory formation and recall. Our observations confirm that isoflurane increases apoptosis and inflammatory responses in the neonatal piglet brain but also suggests novel additional mechanisms by which isoflurane may induce adverse neural and cognitive development by disrupting the expression of genes mediating activity dependent development of neural circuits, the predictive adaptive responses of the brain, memory formation and recall.

## Introduction

Exposure of the developing brain to general anesthesia during early infancy may adversely affect its neural and cognitive development. In a range of animal species early developmental exposure to general anaesthesia without surgery, induces an increase in brain cell death [1–6], reductions in neuro and synapto-genesis [7, 8], a disruption to the expression of cognitively salient genes [9, 10] and deficits in cognitive function that persist throughout life [8, 11, 12]. In humans, attempts to definitively link cognitive dysfunction to anesthetic exposure during infancy have yielded ambiguous results. Several studies have suggested that exposure to general anesthesia is associated with an increased risk of adverse cognitive development, a risk which increases following protracted or multiple procedures [13–16]. However, other studies have concluded that there is no association between a single anesthetic exposure and adverse cognitive development [17–19].

We systematically examined the effects of the anesthetic isoflurane (2%, 6h) without surgery on the neonatal male piglet brain. The developing piglet brain demonstrates remarkable neurodevelopmental similarities to that seen in the human infant [20, 21], which has led to extensive use as models of perinatal neural injury. Three previous studies have demonstrated increases in apoptosis following exposure to isoflurane, all have methodological limitations. One, employed a single piglet per group [22], another whilst linking an increased incidence of apoptosis with an escalating dose of isoflurane, did not include a control group [5] and a third did not randomize piglets and employed a lengthy 24h exposure [23]. All three studies used mixed sex piglets, an important consideration when considering putative extra-apoptotic routes mediating cognitive dysfunction as whilst isoflurane increases brain cell death equivalently in both genders, it induces cognitive deficits in males but not females [24], suggesting that there are gender differences in the mechanisms by which neonatal anesthesia mediates cognitive dysfunction. This may be an important consideration for the interpretation of human studies as neonatal anesthesia exposure may be associated with attention deficit hyperactivity disorder [15], a pattern of cognitive development more common in males [25]. We investigated the effects of isoflurane on apoptosis (TUNEL, caspase-3) and microglial activation (IBA-1). We also investigated if the cells undergoing apoptosis were neurons or oligodendrocytes (by double labeling TUNEL with NeuN and OLIG-2). Electrical activity was continuously monitored using electroencephalography. Finally in an attempt to develop alternative hypotheses of how neonatal isoflurane exposure may induce cognitive dysfunction we investigated gene expression in the anterior cingulate cortex using a combination of porcine microarrays and quantitative polymerase chain reaction (qRT-PCR).

## Materials and Methods

### *In-vivo* procedures

All animal experiments were performed under UK Home Office Guidelines [Animal (Scientific Procedures) Act, 1986] and EEC directive 86/609/EEC, all efforts were made to reduce the number of animals used. This study was approved by the UK Home Office and the UCL ethics board. Twelve large white pedigree male piglets with a mean age of 20.5h (±2.21) and a mean (SD) weight of 1.86kg (±0.48) were randomly assigned to one of two experimental groups: isoflurane-midazolam-fentanyl anesthesia as required by Home Office Guidelines (n = 6) or naïve controls (n = 6). Naïve piglets were briefly sedated with intramuscular midazolam (0.2mg kg^-1^) and euthanised within 10mins of arrival to the facility, to prevent social isolation and maternal deprivation stress over an extended 6h time period affecting gene expression patterns. Piglets randomised to the isoflurane group (Abbott, Berkshire, UK) were sedated with intramuscular midazolam (0.2mg kg^-1^) and initially received isoflurane 2.5–3% v/v via a facemask prior to endotracheal intubation (Portex endotracheal tube, Smiths Medical, Ashford, Kent, UK) and anesthesia with 2% isoflurane for the remainder of the experiment (isoflurane continued for 6h following line insertion and intubation), mean (SD) duration of total experimental intervention 6.8h (±0.48). Piglets were mechanically ventilated; ventilator settings were adjusted to maintain partial pressure of oxygen (PaO_2_) and carbon dioxide (PaCO_2_) at 8-13kPa and 4.5–6.5kPa respectively, allowing for temperature correction of the arterial blood sample. Fractional inspired oxygen concentrations were maintained at 21% and arterial oxygen saturation was monitored throughout by pulse oximetry (Nonin Medical, Plymouth, MN, UK).

An umbilical venous catheter was inserted for infusion of maintenance fluids (10% dextrose, 60ml/kg/day), fentanyl (Mercury Pharma, Co Dublin, Eire, 3mcg kg^-1^ h^-1^) and antibiotic prophylaxis (single doses of benzylpenicillin 50mg kg^-1^ (Genus Pharma, Berkshire, UK,) and gentamicin 2.5mg kg^-1^ (Pathion, Wiltshire, UK)). An umbilical arterial catheter was inserted for continuous monitoring of heart rate and arterial blood pressure. Hourly arterial blood samples were taken to measure PaO_2_, PaCO_2_, pH, electrolytes, urea, creatinine, glucose, lactate and blood haematocrit (Table 1). Arterial lines were maintained by infusing 0.9% saline solution (0.3ml h^-1^); heparin sodium was added at a concentration of 1 IU ml^-1^ to prevent line blockage. All animals received continuous physiological monitoring and intensive life support throughout experimentation. Bolus infusions of crystalloid (0.9% saline, 10ml kg^-1^) and dopamine (5-15mcg kg^-1^ min^-1^) were given, where necessary, to maintain mean arterial blood pressure >40mmHg.

**Table 1:** Physiological variables for piglets at 1h intervals during the 6h exposure to isoflurane anaesthesia.

*Variable*	Time following anesthesia induction						
	Baseline	1 hour	2 hour	3 hour	4 hour	5 hour	6 hour
***Heart rate***	152(16.7)	149(19.2)	156(19.5)	156(21.7)	157(20)	172(26.5)	184(48.6)
***MABP*, *mmHg***	46 (7.6)	52(4.5)	50(6.6)	47(8)	42(5.1)	39(4.8)	43 (4.8)
***Rectal temp*,** ^***o***^***C***	38.6(0.7)	38.2(0.8)	38.5(0.8)	38.4(0.6)	38.1(0.4)	38.3(0.3)	38.2(0.3)
***PaO***_***2***_**, *kPa***	10.7(5)	11.4(1.4)	14.1(3.9)	11.5(0.8)	11.4(3.5)	9.2(1.1)	10.7(1.8)
***PaCO***_***2***_**, *kPa***	5.0(0.9)	6.2(2.0)	7.2(2.3)	7.1(0.4)	7.6(1.3)	7.0(0.7)	6.5(0.6)
***pH***	7.5(0.04)	7.43(0.07)	7.39(0.17)	7.35(0.07)	7.38(0.08)	7.54(0.23)	7.43(0.06)
***BE*, *mmol/l***	6.4(3.6)	5.3(4.5)	6.2(4.6)	4.5(7.8)	7.8(2.1)	4.3(5.7)	6.5(5.7)
***Lactatemmol/l***	3.10(0.9)	3.36(1.4)	3.09(1.0)	1.63(0.7)	2.93(1.2)	1.93(0.5)	3.17(1.3)
***Glucose*,*mmol/l***	4.4(0.6)	6.8(1.3)	7.1(1.0)	8.2 (1.3)	7.7(1.6)	7.6(1.7)	7.9(1.1)
***Calcium*,*mmol/l***	1.43 (0.13)	1.46(0.06)	1.50(0.07)	1.55(0.07)	1.38(0.06)	1.53(0.06)	1.35(0.30)

Mean (SD) are presented for each group. One-way analysis of variance and a repeated measures test were carried out on comparisons across 1 hour intervals. Exposure to isoflurane anaesthesia did not induce significant variation in the physiological variables.

### Histology and immunohistochemistry

Piglets were euthanized with pentobarbital, brains perfused via the cardiac circulatory system with phosphate buffered saline, followed by 4% phosphate buffered paraformaldehyde and post-fixed in 2% paraformaldehyde in PBS (all at pH 7.4 and 4°C) for 10 days. Rostro-caudal coronal slices (5mm thick) of the whole right hemisphere were embedded in paraffin and sectioned to 5μm thickness. Sections were stained with haematoxylin and eosin (H&E) to assess potential neuronal damage and provide a clear visual neuroanatomical template. To assess cell death, sections were stained for DNA fragmentation using terminal deoxynucleotidyl transferase-mediated deoxyuridine-triphosphate (d-UTP) nick-end labelling (TUNEL) histochemistry, and for the appearance of cleaved caspase-3 (CCasp-3) immunoreactivity. Glial activation was assessed by staining for microglial ionized calcium binding molecule 1 (Iba-1) and astrocyte glial fibrillary acidic protein (GFAP) immunoreactivity. Two sections approximate to Bregma levels 00 and -2.0 (5mm apart) were assessed for each animal for each stain.

In brief, TUNEL sections were pre-treated for 15mins in 3% hydrogen peroxide, subjected to a 15mins peptidase pre-digestion with 20μg ml^-1^ protease K (Promega, Southampton, UK) at 65°C and incubated for 2h at 37°C with TUNEL solution (Roche, Burgess Hill, UK). TUNEL was visualized using avidin-biotinylated horseradish complex (ABC, Vector Laboratories, Peterborough, UK) and diaminobenzidine/H_2_O_2_ (DAB, Sigma, Poole, UK). For immunohistochemistry, sections were rehydrated, heat treatment used for antigen retrieval and following blocking with appropriate serum together with 0.1% Triton in PBS, sections were incubated with primary antibody overnight at 4°C: CCasp-3 (1:250: Cell Signalling, New England Biolabs, Herts, UK), Iba-1 (1:1000; Wako, Osaka, Japan) or GFAP (1:1000, Invitrogen, Paisley, UK). Sections were incubated with a biotinylated secondary antibody (1:250) and staining was visualised using ABC (both Vector Laboratories, Camb, UK) and DAB (Sigma, Poole, UK). Iba-1 and CCasp-3 sections were counterstained with cresyl violet. TUNEL and immunohistochemistry sections were dehydrated and cover-slipped with DPX (VWR, Leighton Buzzard, UK).

Fluorescent immunohistochemistry was performed as previously described [26]. In brief, sections were handled as for TUNEL but double labelled with the following primary antibodies: Neuronal nuclear antigen (NeuN, 1:250; Millipore, UK) or Oligodendrocyte transcription factor 2 (Olig2, 1:500; Millipore) following the completion of the TUNEL protocol. TUNEL conjugated biotin residues were detected with Texas Red-Avidin and NeuN/Olig2 with AlexaFluor488-conjugated goat Ig secondary, and donkey anti-goat Ig tertiary antibodies. In addition, sections were doubled labelled with CCasp-3 (1:250, Abcam, Uk) and NeuN or Olig2 (as above). The sections were then covered with DAPI containing aqueous mounting media (Vector Labs, UK).

### Quantification of cell death and glial activation

**Fig 1:**
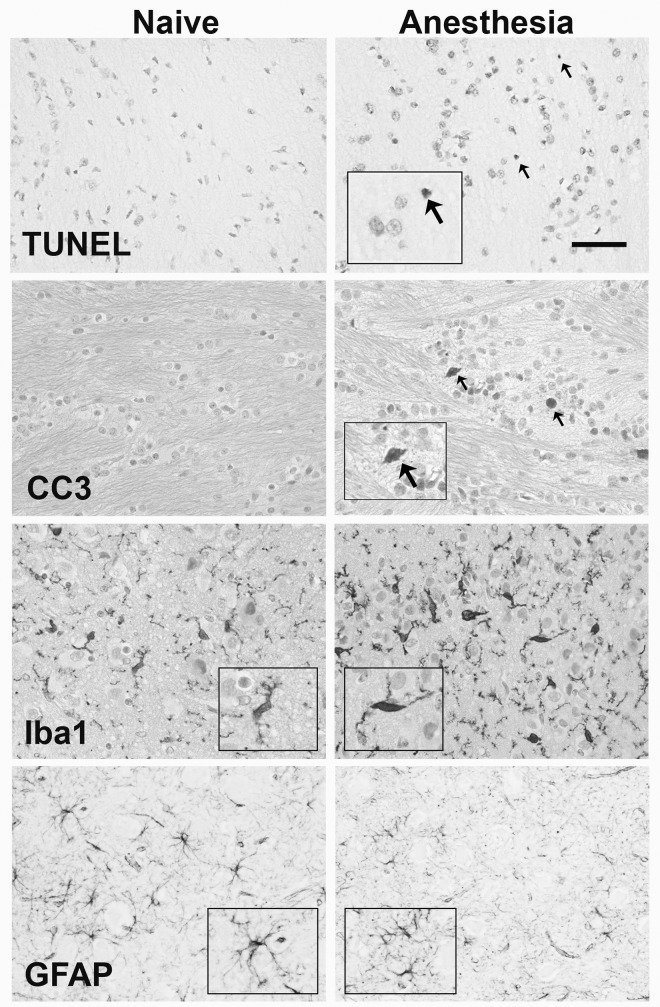
Histology of TUNEL, CCasp-3, Iba-1 and GFAP. Representative photomicrograpths of TUNEL and CCasp-3 in the external capsule and the Iba-1 and GFAP in the thalamus of naïve (left column) and isoflurane exposed (right column) piglets. Arrows indicate TUNEL and CCasp-3 positive cells. CCasp-3 and Iba-1 are counterstained with cresyl violet. Inserts highlight cell activation and morphology (scale bar = 50μm).

**Fig 2:**
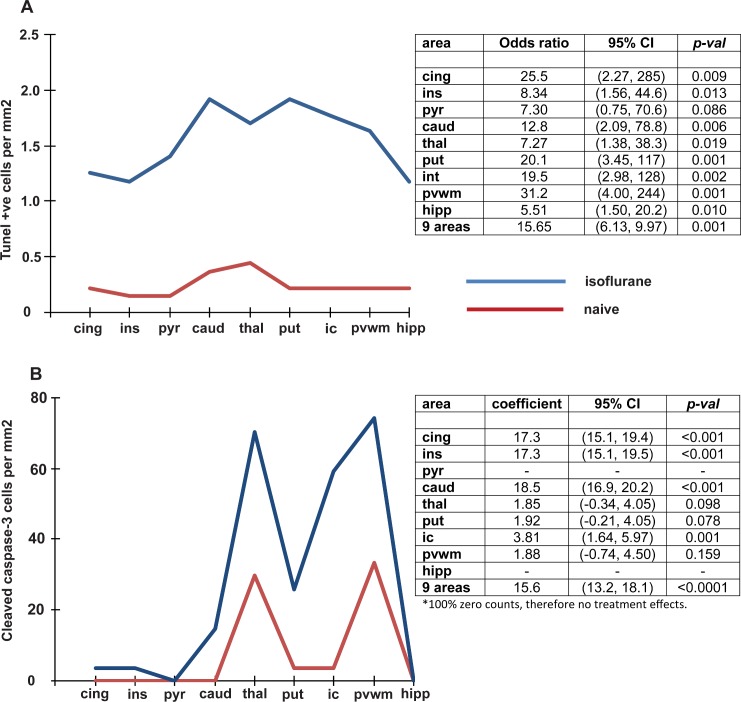
**Effect of a six hour exposure of isoflurane on (A) TUNEL positive cells, (B) Cleaved caspase 3.** The figures on the left show the values relative to brain region in the isoflurane and naïve groups. The tables on the right show the odds ratio (95% CI) and p values for the comparisons between isoflurane exposure and naïve brain in all brain regions as well as the total for the combined 9 regions. Abbreviations; cing = cingulate cortex, ins = insular cortex, pyr = pyriform cortex, caud = caudate nucleus, thal = thalamus, put = putamen, ic = internal capsule, pvwm = periventricular white matter, hipp = hippocampus.

Quantification was undertaken by two independent investigators blinded to treatment group. Recorded values were pooled and means used for analysis. Nine brain regions were assessed for each section: cingulate cortex, insula cortex, pyriform cortex, thalamus, caudate, putamen, periventricular white matter (pvwm), internal capsule and hippocampus (Figs 1 and 2). For each animal, section and brain region, all cells with TUNEL-positive nuclei were counted in three non-overlapping fields of view at x40 magnification (sampling area of 0.0167 mm^2^ per field). Cells expressing cleaved caspase-3 were counted in three non-overlapping fields of view at x40 magnification (0.0167 mm^2^ per field); CCasp-3-labeled cells showing condensed or fragmented nuclei were defined as degenerating cells.

GFAP expression was assessed via densitometry analysis as previously described [26]. In brief, under identical lighting conditions for all sections, two images per brain region were taken at x20 magnification and using image J the total pixel intensity of specific staining was determined.

The activation state of Iba-1 positive cells was assessed by scoring the process number, process complexity (primary, secondary and tertiary) and relative intensity of the soma staining from 0 to 4 (score 0, thin process with tertiary branches and able to visualise the cresyl violet counterstain through the Iba-1 of the soma; 1, tertiary processes but increased staining intensity of the soma; 2, reduced numbers of tertiary processes and thickening of secondary processes with intense enlarged soma; 3, loss of secondary processes and short thickened primary with intense soma; 4, no process and intensely stained soma). Scores were taken of four cells per FOV at x20 magnification (at set positions from the corners of the image) for a total of 24 cells per region per animal. Two investigators, blinded to treatment group, scored each image independently and their scores were averaged.

### Sample preparation and RNA preparation

At euthanasia a fresh, unperfused biopsy of the right anterior cingulate cortex (adjacent to the sulcus at 5mm posterior to bregma) was taken, placed in RNAlater solution (Qiagen, West Sussex, UK), frozen in liquid nitrogen and stored at -80°C until processing (n = 6 naïve, n = 6 anesthesia). RNA was extracted using the standard protocol for animal tissues supplied with the RNAeasy Midi kit (Qiagen, West Sussex, UK). RNA used for microarray was assessed using a Nanodrop spectrophotometer (NanoDrop, Wilmington, DE, USA) and Agilent 2100 Bioanalyser (Agilent, Santa Clara, CA, USA) and all samples had a spectral 260/280 ratio of between 2.05–2.13, and a RIN of 9.9–10. For each sample 200ng of total RNA was amplified and labelled using an Ambion WT expression kit (Invitrogen, Life Technologies Ltd, Paisley, UK). Briefly total RNA is converted to cDNA and then linearly amplified to create an antisense cRNA library. This is then converted to single strand sense cDNA, which is fragmented and end-labelled before hybridisation using a Gene Chip WT terminal labelling and controls kit (Affymetrix, California, USA). The amplified targets were hybridized to Gene Chip Porcine Genome Arrays (Affymetrix, California, USA) overnight and scanned using Gene-Chip Scanner 3000 7 G. Data files were extracted from the image files automatically by Gene-Chip Command Software (version 2, Affymetrix, California, USA) and the CEL file format was subsequently used for analysis.

### Microarray analysis

Analysis was performed using Genespring GX12 (Agilent, California, USA). Data from the individual microarray chips was first normalized across dataset and summarized using the Robust Multi-array Analysis (RMA) algorithm. Data was filtered, as per normal array analysis protocols, to include only those probe sets falling between the 20^th^ and 100^th^ percentile after normalization. One piglet was excluded from analysis for quality control at this point as hybridization to its individual microarray was considered to be faulty.

Initial analysis with Genespring GX12 probe sets detected a total of 79 gene probe sets. Seventeen out of 79 (21%) probe sets obtained lacked a specific gene identity, due to poor characterization of the porcine genome. We identified unknown probe sets using Basic Local Alignment Search Tool (BLAST) and BLAST-Like Alignment Tool (BLAT) sequence alignment software against the pre-labelled mouse genome. We identified a further 15 probe sets but the identity of 2 probe sets remained obscure and these were excluded from further analysis. Ingenuity Pathway Analysis software (IPA; Ingenuity Systems, California, USA) was used to assign the 77 identified genes to a range of known biological functions and metabolic or signalling pathways. Manual labelling of probe sets was also required as IPA software primarily relies on Affymetrix rodent probe annotation numbers to assign biological function.

### Quantitative reverse transcription polymerase chain reaction

Fresh brain tissue collected for microarray analysis was used for qRT-PCR. Primers were designed specifically using the Sus scrofa Ensembl database. The reference genes 14-3-3 protein zeta/ delta (YWAHZ) and ribosomal protein L4 (Rpl4) were chosen to standardize all quantitative experiments. Each sample was assayed in duplicate, and we used the geometric mean of the calculated specific ratio of the gene of interest and for each reference gene.

### Electroencephalography

After endotracheal intubation, multichannel 6 lead EEG (Nicolet, Care Fusion, Wisconsin, USA) monitoring was continued throughout the experimentation as data suggests that isoflurane induced apoptosis may be related to seizure activity [11]. The 6-lead EEGs were reviewed for seizure activity by a clinician familiar with their use in human neonates (Jane Hassel). The five background pattern classification system of de Vries and Hellstrom-Westas [27] was used to describe the amplitude-integrated EEG traces for each piglet [27]. Score 4 = Continuous normal voltage pattern (continuous trace, upper margin >10μV, lower margin >5μV); 3 = Discontinuous normal voltage (discontinuous trace with lower margin intermittently <5μV); 2 = Burst suppression; 1 = Continuous low voltage (upper margin <10μV, lower margin <5μV); 0 = Flat trace (upper and lower margins <5μV).

### Statistical analysis

Thresholds for statistical significance was p<0.05 for all parameters.

**Physiological parameters:** These were examined using repeated measures ANOVA.

**TUNEL analysis:** In each brain region, cells were counted in 3 different fields; the data is therefore clustered with repeated measurements for each pig/ region and the analysis reflected this structure. An initial Chi squared test for association between the cell count and the treatment group (which do not adjust for repeated measures) revealed strong associations between cell count and treatment group in the cingulate, caudate, putamen, internal capsule and pvwm regions and weak associations in the insula and thalamus. Multilevel ordinal logistic regression models (proportional odds models) were fitted to the data, with fixed main effects for treatment group and brain region, and a random pig effect to model the correlations between the correlations in each pig/ region and therefore derive valid confidence intervals and *p*-values. An interaction term between group and region was fitted to determine whether or not the treatment was similar in all regions. The proportional odds assumption was tested by fitting logistic regression models to each pair of dichotomised variables and was found to hold, as similar odds ratios for the treatment effect were obtained in each model. Separate ordinal regression models were fitted to the data from each region, again with a random pig effect.

**Caspase-3 analysis:** The pyriform cortex and hippocampus had 100% zero counts and therefore no treatment effect could be estimated in these areas when separate models are fitted. A mixed effects model with an interaction term between treatment group and area and several of the mixed effects models was fitted in individual areas did not converge. Because of this, a single level ordinal regression model was fitted using data from all areas and including a group by area interaction term. Variances were adjusted for clustering at the pig level due to repeated measures (3 fields). Following on from this, separate single level ordinal logistic regression models were fitted in each area where the treatment effect could be estimated. The results are displayed as coefficients for the treatment effect, adjusted for the area of measurement.

**Iba-1 analysis:** Initial mixed effects ordinal regression models with a random pig effect to account for repeated measures (2 scores in each region) fitted separately to the data from each region showed a similar effect in most areas, with iba-1 counts being higher in the anesthesia group in all but the pvwm regions, where the effect was close to zero. Unsurprisingly then there was no group by area interaction when the data from all regions was fitted in a single model (*p = 0*.*298*). A main effects model was fitted to all data, which returned an estimated treatment effect of 1.95 (95% CI, 0.96, 2.94, *p<0*.*001*) after adjusting for region. Separate models were then fitted to each area separately; consequently the number of observations included in each model is small. For the pyriform cortex, the model did not converge and therefore an individual coefficient from the mixed effects model for this region could not be included. For this region only, therefore, a single level ordinal regression model was fitted, with standard errors, derived confidence intervals and p-values adjusted for clustering at the pig level.

**GFAP analysis:** Data was divided by 10^6^ to make it more usable and to ensure stable modeling. The data was approximately normal, although there were 2 outliers, both in one pig from the anesthesia group in the hippocampus. Because there were two observations from each subject/region combination, linear mixed models were fitted that included a random subject effect to allow for clustering and subsequent effects on the standard errors. A term for the interaction between region and group was initially included in the model, but there was little evidence to support the hypothesis that the treatment effect was substantially different in different regions (*p = 0*.*151*), therefore a main effects model was fitted, with main effects for area and treatment group. Fitting separate models to the data from each region did not show any highly significant results; the largest effect was in the pyriform cortex.

**Microarray data:** Our microarray data due to tailing was not normally distributed, therefore analysis was performed using a one-way ANOVA followed by a Mann-Whitney unpaired post-hoc test and a Benjami-Hochberg FDR multiple testing correction; *p*-values were calculated asymptotically using Genespring GX12.

## Results

### Physiological parameters are not significantly altered during a 6h exposure to isoflurane

There were no intergroup differences in body weight or post-natal age. Baseline physiological (heart rate and mean arterial blood pressure) and biochemical (blood lactate, base excess and glucose) parameters were controlled to within normal values throughout the experiments, with no statistically significant hour-to-hour variation in physiological variables across the 6h period of isoflurane exposure (Table 1). One piglet received dopamine treatment to maintain mean arterial blood pressure above 40mmHg.

### Histology

Representative TUNEL, CCasp-3, GFAP and Iba-1 immunohistochemistry are illustrated in Fig 1. H&E staining demonstrated that there were no macro- or microscopic histopathological differences associated with isoflurane exposure (not shown).

### A 6h isoflurane exposure increases cell death in grey and white matter

Cell death assessed by TUNEL staining is shown in Figs 1 and 2A. In 8 of the 9 regions, cell death in isoflurane-treated piglets was significantly higher than that in the naïve piglets, with the exception of the pyriform cortex. Of note, 83% of the cell counts in the naïve group, but just 31% of those in the isoflurane-treated group, yielded 0 TUNEL-positive cells per field of view. Overall a significant treatment effect was observed following the application of a fitted ordinal regression model. The estimated odds of a higher cell count is 15.65 times higher in the isoflurane treated group (70.04±7.18 mm^-2^, mean (±SEM)) compared to the naïve group (9.88±2.69 mm^-2^) having adjusted for variations between brain regions. This data supports a range of odds intervals between 6.13 and 39.97 (95% CI for the odds ratio (6.13, 9.97)). This is equivalent to a total 7.0 fold increase in the total number of TUNEL-positive cells across nine forebrain regions compared with the naïve group. No significant difference in the cell count was found between different regions of the brain (*p* = 0.121).

Cell death measured by CCasp-3 expression is shown in Fig 2A. Separate single level ordinal logistic regression models demonstrated that isoflurane anesthesia induces significant increases (*p<0*.*05*) in the number of dying cells expressing cleaved caspase 3 in the cingulate and insular cortices, caudate nucleus and internal capsule. Cells expressing CCasp-3 were sparsely distributed, representing 35.7% of the TUNEL-positive cell population when calculated as a percentage of the total number of TUNEL-positive cells (mean 24.69±4.49 CCasp-3 positive cells mm^-2^).

**Fig 3:**
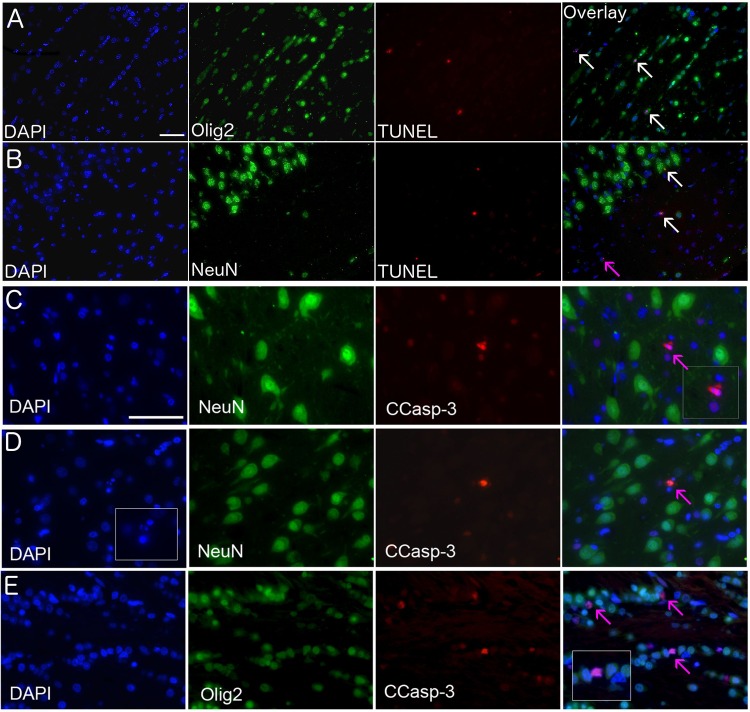
Co-localisation of cell death and lineage markers. TUNEL and CCasp-3 (red), oligodendrocyte cell lineage marker (Olig-2, green), neuronal cell linage marker (NeuN, green), nuclear stain (DAPI, blue). In the (A) periventricular white matter, (B) dorsoparietal cortex layer 1/2, (C) thalamus, (D) sensoriomotor cortex layer 3/4, (E) external capsule. Cells expressing TUNEL and either Olig-2 or NeuN are indicated with white arrows in the overlay panel (A and B), and those not co-labelling (due to loss of cell specific markers induced by the stage of death or alternate cell type) are indicated with a pink arrow (B, C, D). Scale bar in (A-B) is 50μm and in (C-E) is 25μm.

We performed double labelling for markers of cell lineage (Olig2 and NeuN) and cell death (TUNEL and CCasp-3) and were able to co-localise almost all TUNEL-positive cells with either NeuN or Olig2 (Fig 3). Cells failing to co-localise typically had strong TUNEL staining and fragmented nuclei, indicating a late stage of cell death. As previously reported, we were unable to detect Olig2 and NeuN cells co-expressing CCasp-3 [1, 2]. However, we noted CCasp-3 cells adjacent to Olig2 positive cells in the white and grey matter where CCasp-3 positive cells were small and evenly distributed through the cortical layers (Fig 3).

### A 6h isoflurane exposure induces microglial activation

**Fig 4:**
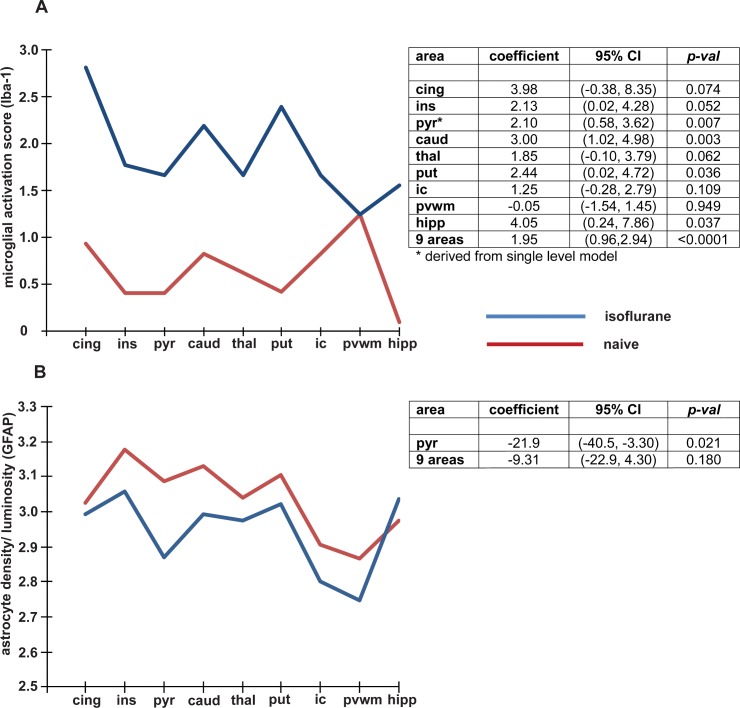
Effect of a six hour exposure of isoflurane on (A) Microglial activation and (B) Astrogliosis. The figures on the left show the values relative to brain region in the isoflurane and naïve groups. The tables on the right show the odds ratio (95% CI) and p values for the comparisons between isoflurane exposure and naïve brain in all brain regions as well as the total for the combined 9 regions. Abbreviations; cing = cingulate cortex, ins = insular cortex, pyr = pyriform cortex, caud = caudate nucleus, thal = thalamus, put = putamen, ic = internal capsule, pvwm = periventricular white matter, hipp = hippocampus.

Analysis of immunostaining for Iba1, a microglial marker, demonstrated a significant increase in microglial activation score in the insula and pyriform cortex, hippocampus, thalamus and caudate (Fig 4A). We also observed that gene expression for the microglial activation marker CD86 was significantly increased using qRT-PCR (S1 Fig). Mixed effects ordinal regression models, accounting for repeated measures (2 scores in each region) fitted separately to the data from each region, demonstrated that microglial activation (iba-1 counts) showed a similar effect in most areas, with iba-1 counts being higher following isoflurane exposure in all but the pvwm where the effect was close to zero. Unsurprisingly then there was no group by area interaction when the data from all regions was fitted in a single model (p = 0.2984).

### A 6h isoflurane exposure induces a reduction in astrogliosis

Analysis of immunostaining for GFAP, a cytoskeletal protein found exclusively in astrocytes, indicated a significant decrease in staining in the pyriform cortex only (Fig 4Bd). Examining the data in each region separately, the mean luminosity was lower in the isoflurane treated group compared to the control group in all regions other than the hippocampus, suggesting that isoflurane induces a reduction in astrogliosis.

### A 6h isoflurane exposure induces diverse transcriptional changes

All gene expression data are reported as fold-change from the naïve data. Empirical analysis of function using database information indicated that 26 of the 77 affected genes coded for genes with known function in the regulation of transcription; 13/26 (50%) of these transcription factors were down-regulated (Table 2). We also identified 23 transcripts that are important for the mediation of neural plasticity, long term potentiation/depression, memory formation and recall; 15/23 (65%) of these transcripts were down-regulated (Table 3). All 77 gene transcripts are included online as S1 Table. Analysis of the 77 affected genes to identify known canonical pathways induced by 6h of isoflurane exposure failed to demonstrate any statistically significant biologically relevant pathways. Using the targeted approach of qRT-PCR, we further validated that gene expression following 6h isoflurane exposure was significantly reduced for dual specificity phosphatase 4 (DUSP-4), and increased for early growth response protein 1 (EGR1) and the microglial activation marker CD86 (S1 Fig).

**Table 2:** Changes in the expression of genes thought to be important for the control of gene transcription following a 6h isoflurane exposure.

Fold change	Gene name (*abbreviation*)
-3.30	Neuronal PAS domain protein 4 (*NPAS4*)
-2.09	Early Growth Response Protein 1 (*EGR-1/zif-268*)
-2.05	Early Growth Response protein 4-like (*EGR-4*)
-2.02	Identical to Early Growth Response 1 protein (*EGR-1/zif-268*)
-1.79	Adenylate cyclase activating polypeptide 1 (pituitary) (*ADCYAP1*)
-1.76	Neuron-derived Orphan Receptor-1α (*NOR-1*)
-1.65	sSNORD 14B snoRNA (*SNORD 14B*)
-1.52	Histone deacetylase 9 isoform 5 or 6 (*HDAC9*)
-1.56	Zinc finger protein 804B (*ZNF804B*)
-1.51	Spermatogenesis-associated, serine-rich 2-like (*SPATS2L*)
-1.51	STAM binding protein-like 1 (*STAMBPL1*)
-1.51	F-box protein 43 (*FBXO43*)
-1.50	ELAV (embryonic lethal, abnormal vision, Drosophila)-like 2 (Hu antigen B) (*ELAVL2*)
2.20	DDB1 and CUL 4 associated factor 4 (*DCAF4*)
1.88	microRNA let 7c stem loop (*MIRLET 7c*)
1.69	Histone H2A type 1 (*HIST1H2AA*)
1.65	BTG family, member 2 (*BTG9*)
1.62	High mobility group box 2 (*HMGB2*)
1.62	GMP synthase (glutamate hydrolase) (*GMPS*)
1.59	HORMA domain containing 1 (*HORMAD1*)
1.59	Thioredoxin-like protein 4B-like (*TXNL4B*)
1.57	Ras-like protein family member 10A-like (*RASL10A*)
1.55	D site-Binding Protein-like (*DBP*)
1.53	Ribosomal protein S6 kinase α5-like (*RPS6KAS*)
1.51	MEF2-activating motif and SAP domain-containing transcriptional regulator-like (*MAMSTER*)
1.50	Zinc finger matrin-type protein 1-like (*ZMAT 1*)

**Table 3:** Changes in the expression of gene transcript thought to be important in the mediation of neural plasticity, long term potentiation, learning and memory, following a 6h isoflurane exposure.

Fold change	Gene name (*abbreviation*)
-3.30	neuronal PAS domain protein 4 (*NPAS4*)
-2.80	cycloxygenase 2 (aka prostraglandin-endoperoxide synthase 2) *(COX-2)*
-2.23	brain derived neurotrophic factor *(BDNF)*
-2.09	early Growth Response Protein 1 (*EGR-1/zif-268*)
-2.05	early Growth Response protein 4-like (*EGR-4*)
-2.05	Dual specificity protein phosphatase 4 (*DUSP-4)*
-2.02	identical to Early Growth Response 1 protein (*EGR-1/zif-268*)
-1.94	adenylate cyclase type 8-like *(ADCY8)*
-1.92	leucine rich and Ig domain containing 2 *(LINGO2)* (2 identical transcripts)
-1.92	leucine rich and Ig domain containing 2 *(LINGO2)*
-1.84	5-hydroxytryptamine receptor 1A like
-1.79	adenylate cyclase activating polypeptide 1 (pituitary) (*ADCYAP1*)
-1.56	zinc finger protein 804CB *(ZNF804B)*
-1.50	ELAV (embryonic lethal, abnormal vision, Drosophila)- like 2 *(ELAVL2)*
-1.50	protein FAM19A1-like *(FAM19A1-like)*
4.09	transcript with identical homology to the ephrin type A receptor A3 *(EPHRA3)*
3.10	transcript with identical homology to the ephrin type A receptor A3 *(EPHRA3)*
3.10	HEAT repeat containing protein 3 *(HEATR3)*
1.88	microRNA let-7c stem loop *(MIRLET7C)*
1.69	arrestin domain containing 3 *(ARRDC3)*
1.65	BTG family, member 2 *(BTG2)*
1.56	immunoglobin superfamily containing leucine-rich repeat 2 *(ISLR2)*
1.52	synaptotagmin 17 *(SYT17)*

### A 6h isoflurane exposure to isoflurane is not associated with seizure activity

**Fig 5:**
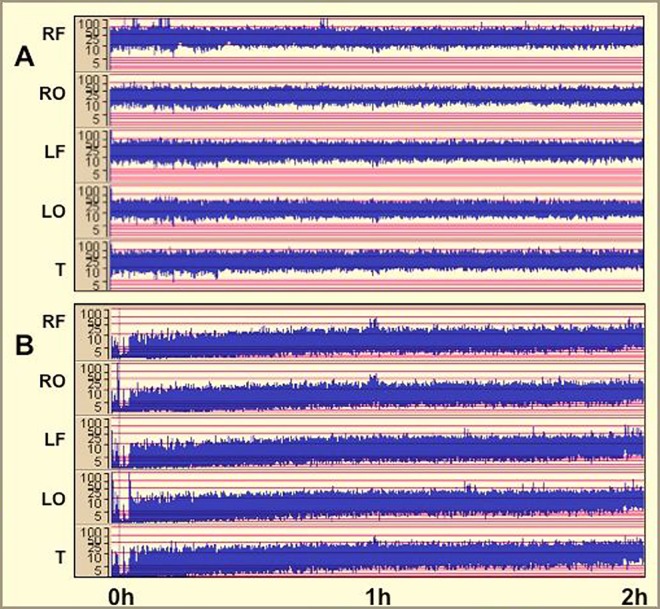
Representative amplitude-integrated electroencephalographs (aEEGs) in response to 2h of isoflurane exposure (0 to 2h). **A.** Continuous normal voltage (CNV) throughout. **B.** Low voltage, recovering to CNV by 2h.

No seizures were identified on multichannel EEG in any piglet throughout anesthesia. Following analysis of amplitude-integrated EEG (aEEG) and using the pattern recognition classification [27], three piglets initially had low voltage before recovering to continuous normal voltage (CNV) (all within 2 hours) and four piglets had CNV throughout the experiment (Fig 5 and Table 4). Although numbers were small, there was no apparent difference in cell death in the three piglets with initial abnormal aEEG compared to those with normal aEEG throughout (data not shown).

**Table 4:** Amplitude-integrated electroencephalography. Findings for each piglet were scored using the de Vries and Hellstrom-Westas [27] pattern classification. Three out of six piglets initially had a suppressed aEEG trace. All piglets had a normal aEEG trace by 1h. No piglet had seizures on 6-lead EEG. CNV: continuous normal voltage; DNV: discontinuous normal voltage; BS: burst suppression.

*Piglet number*	Time following anesthesia induction							
	10 min	30 min	1 hour	2 hour	3 hour	4 hour	5 hour	6 hour
***1***	CNV	CNV	CNV	CNV	CNV	CNV	CNV	CNV
***2***	BS	DNV	CNV	CNV	CNV	CNV	CNV	CNV
***3***	DNV	CNV	CNV	CNV	CNV	CNV	CNV	CNV
***4***	DNV	CNV	CNV	CNV	CNV	CNV	CNV	CNV
***5***	CNV	CNV	CNV	CNV	CNV	CNV	CNV	CNV
***6***	CNV	CNV	CNV	CNV	CNV	CNV	CNV	CNV

## Discussion

This data demonstrates that a 6h exposure to 2% isoflurane and fentanyl (3mcg kg^-1^ h^-1^) induces statistically significant increases in neuronal and oligodendrocyte apoptosis in the male neonatal piglet brain. This increase in apoptosis was accompanied by a significant increase in microglial activation and the microglial activation marker gene CD86. Our observations that a 6h isoflurane exposure induces diverse changes in gene transcripts with known functions in the regulation of transcription (26/77) and the mediation of neural plasticity (23/77) suggests alternative extra-apoptotic mechanisms by which an anesthetic exposure may influence neural and cognitive development.

### A 6h isoflurane exposure induces cell death

Absolute levels of cell death induced by our 6h anesthetic exposure (7-fold increase compared to naïve brains) were low when compared to those seen following similar exposures to isoflurane in post-natal neonatal rodents or the rhesus macaque [rodents; 4, 29, 28, macaques; 1–3]. In the 6d old macaque (which are thought to be neuro-developmentally equivalent to a 4–6 month old human), a 5h exposure to isoflurane resulted in a 12.6-fold increase in cell death [1, 2]. However, the same 5h exposure in a 120d gestation fetal macaque (neuro-developmentally equivalent to a mid-third trimester human) resulted in a 4.1 fold increase in cell death [3]. As the 24h old piglets employed in this study are thought to be neuro-developmentally equivalent to a neonatal human, this may reflect an increase in isoflurane toxicity with increasing neuro-developmental age. Similar effects have been reported in a longitudinal rodent study [28]. One potential confounding factor that may account for the higher levels of cell death observed in the macaque is that the macaques were allowed a 3h recovery (withdrawal of anesthesia and subsequent re-awakening) prior to termination during which more cells may undergo apoptosis [1–3].

We measured cell death using TUNEL (a marker of DNA fragmentation) and a marker more specific to apoptosis, CCasp-3. The CCasp-3 positive cell population accounted for only 35.7% of TUNEL positive cells in our isoflurane exposed brains. Similar results were found comparing CCasp-3 to TUNEL in piglets exposed to 24h isoflurane [23] and comparing CCasp-3 to dying cells identified morphologically with condensed chromatin in P6 macaques exposed to 5h isoflurane [1, 2]. This lack of complete overlap may be because CCasp-3 expression is transient and ceases during early and late stages of apoptosis and that isoflurane accelerates the process of apoptosis that occurs during the normal maturation of the brain [29, 30]. Given the relatively early time point (6h from the onset of isoflurane anesthesia) it is likely that we are observing cell death via both caspase dependent and independent mechanisms [31, 32, 33].

We phenotyped dying cells and co-localized the majority of cells expressing TUNEL with lineage markers for neurons (NeuN) and oligodendrocytes (Olig2) (Fig 3), demonstrating that in the neonatal piglet, anesthesia induces cell death in both neurons and oligodendrocytes. This vulnerability of both neurons and oligodendrocytes to isoflurane exposure has previously been reported in both piglets and macaques [1–3, 23]. We were unable to co-localize CCasp-3 expressing cells with any cell lineage markers, a similar problem was observed by Brambrink in the macaque; this may reflect the fact that cells expressing CCasp-3 are at a later stage of cell death, which results in a diminished expression of lineage markers [1, 2].

### A 6h anesthesia exposure has pro-inflammatory effects

A pro-inflammatory effect of anesthetic exposure, including isoflurane, on the adult and neonatal brain has been previously observed in rodents [10, 34, 35, 36] but anti-inflammatory effects or non-effects are also reported [28, 37]. The effects of isoflurane have included loss of the astrocyte cytoskeletal protein GFAP [38, 39] although we noted this only in one region of the piglet brain (Fig 4B). We observed an increase in microglial activation state based on morphology (Fig 4A) and an increase in the expression of CD86, a prototypical marker of microglial activation. It is not clear whether this marginal effect on astrocytes or the type of microglial activation is damaging or reflects the activation of an endogenous protective response [40].

### A disruption to the expression of genes important for transcriptional regulation and neural plasticity suggests novel mechanisms by which isoflurane may affect neural and cognitive development

Our observations suggest that a 6h isoflurane exposure induces significant changes in the expression of 26 gene transcripts important for transcriptional regulation **(**Table 2, S1 Table) and 23 gene transcripts important for neural plasticity, long-term potentiation and memory (Table 3, S1 Table); these pathways suggest alternative extra-apoptotic mechanisms by which neonatal anesthesia exposure may induce cognitive impairments.

Using microarray we observed a statistically significant 2.2 fold reduction in the expression of BDNF, although when using qRT-PCR this reduction did not reach statistical significance. BDNF is a critical regulator of neuronal plasticity following activity dependent neural activation, and also has roles in neuronal survival, oligodendrocyte maturation and myelination. Previous studies have demonstrated that a single isoflurane exposure induces a reduction in BDNF transcription and cognitive deficits in adult rats [4, 9, 41, 42]. We also observed robust reductions in the expression of a number of other genes important for the mediation of LTP and the formation of memory, including neural pas domain 4 (NPAS-4, -3.3 fold), which regulates the excitatory-inhibitory balance within neuronal circuits via BDNF signaling [43, 44], cyclooxygenase-2 (COX-2, -2.80), which is necessary for the induction of LTP and spatial learning [45] and early growth response protein (egr-1/ zif-268, -2.09) [46–48]. Interestingly there was a significant increase in the expression of two transcripts almost identical to the ephrin type receptor A3 (+4.09, +3.10). Ephrin A3 signalling is required for the induction of LTP and the control of dendritic spine morphology but also lowers glutamate transporter expression, which can result in glutamate exitotoxicity [49, 50].

The developing mammalian brain can demonstrate remarkable plastic and adaptive responses many of which serve to adapt the brain to accurately interpret and interact with the environment that it will encounter when mature. Our data suggests that a 6h neonatal exposure to isoflurane disrupts the expression of genes important for this process during a crucial period of neural development, which may affect neural and cognitive development in three inter-dependent ways, by affecting the normal activity dependent development of neural circuits, by interfering with evolutionary processes designed to ensure that a developing brain is optimally adapted to the environment that it will encounter when mature and by directly disrupting the expression of genes supporting the specialised form of neural plasticity that stores information, learning and memory [51, 52].

Immediately before birth, co-ordinated cell type-specific epigenetic and transcriptional remodelling begins to enable the brain’s adaptation from an intrauterine to extrauterine environment. This facilitates rapid postnatal synaptogenesis and neocortical expansion and allows the integration of activity-dependent neuronal gene expression and development with salient environmental and developmental cues. The most intense period for this epigenetic and transcriptional shift extends from just before birth to the first year of life in the human [53–55]. Activity-dependent neuronal gene expression, remodelling and maturation during early development is important for the proper developmental function of neural circuits. In the extensively studied visual, auditory and attention systems, disruption of this process can result in lifelong cognitive deficits and a delay in the maturation of the neural systems sub-serving these functions [51, 52, 56–59]. Many these processes can be incorporated into variants of the predictive adaptive response hypothesis which provides a mechanistic framework by which a developing organism can attempt to predict or modify its neural phenotype to optimally interact with its environment when mature [60–63]. These predictive adaptive responses generally have advantageous properties and a key prediction of this hypothesis is that a brain, which has during development successfully predicted its adult environment will interpret its surroundings in an optimal manner which benefits the organism by increasing its survival and Darwinian fitness. If this process is disrupted the resultant predictive adaptive responses may have contingent or dichotomous properties which may lead to a mismatch between a neural phenotype and its environment. One of the most studied examples is the relationship between maternal nutrition and offspring, metabolism and behaviour, but elements of this hypothesis have been incorporated into theories that explain the development of a range of neural phenotypes that are characterised by poor attentional and inhibitory control [60–67].

Our data does raise questions, does the expression of these genes return to their pristine state following the withdrawal of anesthesia, or is their expression disrupted throughout an organism’s lifespan by epigenetic mechanisms? The expression of BDNF protein is thought to return to its pre-exposure value within 7–14 days [9], but it is thought that an enriched environment is necessary to return levels of BDNF gene transcription to its pre-exposure level [10, 68]. There is no available data as to what is the time period is for the expression of other genes that mediate neural plasticity to return to their pristine state, but an extended disturbance of their expression patterns during development is likely to have deleterious effects on neural and cognitive function by interfering with the mechanisms described above.

### 6h anesthesia is not associated with seizure activity

We did not observe any seizure activity in our isoflurane exposed piglets (Table 4 and Fig 4). This contrasts with data from neonatal rodent studies, which suggest that isoflurane and sevoflurane increase neuronal cortical activity leading to seizure like EEG patterns [11, 69]. In both our piglet and previous macaque studies [1–3] there is considerably less cell death than that seen in rodents following a similar exposure to isoflurane, which may be partly explained by the absence of seizure activity. Likewise EEG studies in anesthetised humans have not reported seizures, however burst suppression is frequent [70–72]. Whether burst suppression exerts pathological effects is unclear. We identified burst suppression in 1/6 and discontinuous voltage in 3/6 piglets in our study. In a recent clinical study of sevoflurane exposure there was little anesthesia-dependent change in EEG in infants aged 0–5 months and only from age 6 months did the typical anesthesia-dependent EEG patterns seen in older children emerge [72]. As such, although we did not observe substantial changes in EEG, these may only become apparent in older subjects as seen in this human study.

### Caveats associated with anesthesia administration in our study

The minimum alveolar concentration of isoflurane to achieve anesthesia has been reported as 1.5 +/- 0.21% in neonatal piglets [73]. Following a pilot dose response study, we employed an anesthetic dose of isoflurane (2%), midazolam (0.2 mg kg^-1^) and fentanyl (3 mcg kg^-1^ h^-1^), which provided sufficient anesthesia to prevent movement following a skin incision in the absence of a muscle relaxant. Similar isoflurane doses have been used in neonatal (<24 hour-old) and infant (4 week-old) swine models [23, 74]. These studies found that mean arterial blood pressure varied significantly with anesthesia. Although we did not find significant changes in mean arterial blood pressure, one animal required dopamine to support its blood pressure, indicating anesthetic exposure may induce significant cardiovascular sequelae in neonatal piglets. Furthermore it is reported that isoflurane impairs cerebral auto-regulation in large animal models and human infants leading to increased cerebral blood flow, an effect that is independent of mean arterial pressure [37, 74, 75].

### Conclusion

This study demonstrates that a 6h isoflurane exposure to the neonatal piglet brain is associated with significant increases in apoptosis of both neurons and oligodendrocytes. This is accompanied by an increase in microglial activation and the expression of its associated activation marker CD86. These effects have been previously described in a range of species and have led to the development of hypotheses that anesthesia induces adverse neural and cognitive development by the induction of developmentally inappropriate apoptosis and inflammation. However, our observations that isoflurane induces the disruption to the expression of 26 gene transcripts important for transcriptional regulation (Table 2) and 23 gene transcripts important for the mediation of neural plasticity (Table 3) during an important period for the environmental and activity dependent development of neural circuits, suggests that isoflurane also exerts adverse effects on neural and cognitive development by a direct action on genes important for neural plasticity, a process that may occur in parallel with apoptotic or inflammatory mechanisms. Our work suggests the need for longitudinal studies of gene expression following recovery from anesthesia to determine if and when the expression of genes supporting neural plasticity return to their developmentally normal pre-exposure levels. The neonatal piglet model closely parallels human neurodevelopment, incorporates clinically relevant continuous intensive care support and monitoring and provides a, experimental platform for trialling therapeutic interventions to ameliorate the negative neurotoxic consequences of anesthesia.

## Supporting Information

S1 Fig qRT-PCR gene expression data. For brain derived neurotrophic factor (BDNF), dual specificity phosphatase 4 (DUSP 4), early growth response protein 1 (EGR1 aka zif-268), neuronal PAS domain protein 4 (NPAS4) and cluster of differentiation (CD86). *p<0.05 following a t-test.
(DOCX)

S1 Table Disruption in the expression of 79 gene transcripts were responsive to 6h isoflurane exposure. 41 transcripts were up-regulated and 38 were down-regulated. A gene was considered to have been responsive to 6h isoflurane exposure if it’s relative expression changed by a factor of 1.5 fold or above. *p*-vals were obtained using one-way ANOVA followed by Mann-Whitney unpaired post-hoc test with Benjamini-Hochberg FDR multiple testing correction, using the internal software of Genespring GX12
(DOCX)
