# Supplementary material for: Isoflurane Exposure Induces Cell Death, Microglial Activation and Modifies the Expression of Genes Supporting Neurodevelopment and Cognitive Function in the Male Newborn Piglet Brain
Source: PLoS One. 2016 Nov 29;11(11):e0166784. doi: 10.1371/journal.pone.0166784 (PMC5127656; doi:10.1371/journal.pone.0166784)
Supplement: S1 Fig — For brain derived neurotrophic factor (BDNF), dual specificity phosphatase 4 (DUSP 4), early growth response protein 1 (EGR1 aka zif-268), neuronal PAS domain protein 4 (NPAS4) and cluster of differentiation (CD86). *p<0.05 following a t-test. (DOCX) [file pone.0166784.s001.docx]

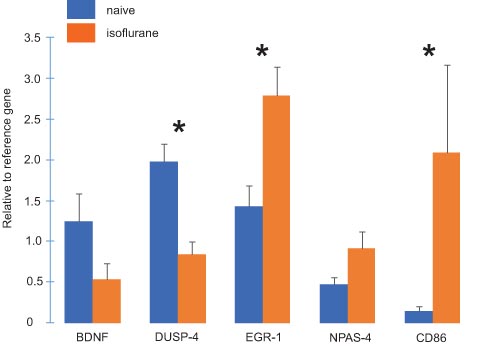
 **Supplementary Figure 1.** qRT-PCR gene expression data for brain derived neurotrophic factor (BDNF), dual specificity phosphatase 4 (DUSP 4), early growth response protein 1 (EGR1 aka zif-268), neuronal PAS domain protein 4 (NPAS4) and cluster of differentiation (CD86). *p<0.05 following a t-test.
