# Supplementary material for: Isoflurane Exposure Induces Cell Death, Microglial Activation and Modifies the Expression of Genes Supporting Neurodevelopment and Cognitive Function in the Male Newborn Piglet Brain
Source: PLoS One. 2016 Nov 29;11(11):e0166784. doi: 10.1371/journal.pone.0166784 (PMC5127656; doi:10.1371/journal.pone.0166784)
Supplement: S1 Table — 41 transcripts were up-regulated and 38 were down-regulated. A gene was considered to have been responsive to 6h isoflurane exposure if it’s relative expression changed by a factor of 1.5 fold or above. p-vals were obtained using one-way ANOVA followed by Mann-Whitney unpaired post-hoc test with Benjamini-Hochberg FDR multiple testing correction, using the internal software of Genespring GX12 (DOCX) [file pone.0166784.s002.docx]

**Supplementary Table 1.**

The expression of 79 gene transcripts were responsive to 6h isoflurane exposure; 41 were up-regulated and 38 were down-regulated. A gene was considered to have been responsive to 6h isoflurane exposure if it’s relative expression changed by a factor of 1.5 fold or above. *p*-vals were obtained using one-way ANOVA followed by Mann-Whitney unpaired post-hoc test with Benjamini-Hochberg FDR multiple testing correction, using the internal software of Genespring GX12

| **Fold change** | **Gene product** | **Abbreviation** |
| --- | --- | --- |
| 4.09 | transcript with identical homology to the ephrin type A receptor A3 | EPHRA3 |
| 3.10 | transcript with identical homology to the ephrin type A receptor A3 | EPHRA3 |
| 3.10 | HEAT repeat containing protein 3 | HEATR3 |
| 2.22 | hemoglobin subunit alpha-like | HBA |
| 2.10 | transcript with identical homology to the DDB1 and CUL4 ASSOCIATED FACTOR 4 | DCAF4 |
| 2.04 | 5-aminolevulinate synthase, erythroid-specific, mitochondrial-like | ALAS |
| 1.88 | microRNA let-7c stem loop | MIRLET7C |
| 1.86 | mitochondrially encoded serine | mtSER |
| 1.78 | microfibrillar-associated protein 2-like | MFAP2 |
| 1.77 | ADP-ribosyl cyclase 2-like | BST1 |
| 1.76 | mevalonate kinase-like | MVK |
| 1.76 | complement component C9 | C9 |
| 1.74 | monocarboxylate transporter 7 | SLC16A6 |
| 1.69 | arrestin domain containing 3 | ARRDC3 |
| 1.69 | histone H2A type 1 | HIST1H2AA |
| 1.68 | lysyl oxidase | LOX |
| 1.66 | hemoglobin, beta | HBB |
| 1.65 | putative zinc finger and SCAN domain containing protein 5D (dubious product pseudogene) | ZSCAN5D |
| 1.65 | BTG family, member 2 | BTG2 |
| 1.64 | StAR-related lipid transfer (START) domain containing 4 | STARD4 |
| 1.63 | unidentified transcript |  |
| 1.62 | cortexin-2-like | Ctxn2 |
| 1.62 | GMP synthase (glutamate hydrolase) | GMPS |
| 1.62 | high mobility group box 2 | HMGB2 |
| 1.59 | HORMA domain containing 1 | HORMAD1 |
| 1.59 | thioredoxin-like protein 4B-like | TXNL4B |
| 1.58 | phosphoinositide-3-kinase, regulatory subunit 3 (gamma) | PIK3R3 |
| 1.57 | ras-like protein family member 10A-like | RASL10A |
| 1.56 | serine palmitoyltransferase, long chain base subunit 3 | SPTLC3 |
| 1.56 | uncharacterized protein KIAA0825-like | KIAA0825 |
| 1.56 | immunoglobulin superfamily containing leucine-rich repeat 2 | ISLR2 |
| 1.55 | alkaline ceramidase 2-like | ACER2 |
| 1.55 | D site-binding protein-like | DBP |
| 1.54 | ras-like protein family member 10A-like | RASL10A |
| 1.53 | MIND kinetochore complex component, homolog (S cerevisiae) | DSN1 |
| 1.53 | ribosomal protein S6 kinase alpha-5-like | RPS6KA5 |
| 1.52 | solute carrier family 16, member 9 (monocarboxylic acid transporter 9) | SLC16A9 |
| 1.52 | synaptotagmin-17 | SYT-17 |
| 1.51 | MEF2-activating motif and SAP domain-containing transcriptional regulator-like | MAMSTER |
| 1.51 | alanine--glyoxylate aminotransferase 2-like 1-like | AGXT2L1 |
| 1.50 | zinc finger matrin-type protein 1-like | ZMAT1 |
| -3.30 | neuronal PAS domain protein 4 | NPAS4 |
| -2.80 | Cyclooxygenase 2 | PTGS2 |
| -2.39 | transmembrane protein 74-like | TMM74 |
| -2.23 | brain-derived neurotrophic factor | BDNF |
| -2.10 | ankyrin repeat domain 34C | ANKRD34C |
| -2.09 | early growth response 1 protein | EGR-1 |
| -2.05 | dual specificity protein phosphatase 4-like | DUSP-4 like |
| -2.05 | early growth response protein 4-like | EGR4 |
| -2.05 | dickkopf-related protein 2-like | DKK2 |
| -2.02 | early growth response 1 protein | EGR1 |
| -1.94 | adenylate cyclase type 8-like | ADCY8 |
| -1.92 | leucine rich and Ig domain containing 2 | LINGO2 |
| -1.92 | leucine rich and Ig domain containing 2 | LINGO2 |
| -1.84 | 5-hydroxytryptamine receptor 1A-like | HTR1A |
| -1.80 | leucine-rich repeat and WD repeat-containing protein like | KIAA1239 |
| -1.79 | adenylate cyclase activating polypeptide 1 (pituitary) | ADCYAP1 |
| -1.78 | tumor necrosis factor inducable gene 6- protein precursor | TNFA1P6 |
| -1.76 | neuron-derived orphan receptor-1 alfa | NOR-1 |
| -1.76 | sprouty-related, EVH1 domain containing 2 | SPRED2 |
| -1.72 | oxysterol binding protein-like 3 | OSBPL3 |
| -1.68 | proline-rich protein 16-like | PRR16 |
| -1.67 | tribbles homolog 2-like | TRB-2 |
| -1.66 | leucine rich repeat transmembrane neuronal 3 | LRRTM3 |
| -1.65 | sSNORD 14B a snoRNA | SNORD 14B |
| -1.61 | cysteine-rich secretory protein LCCL domain containing 1 | CRISPLD1 |
| -1.57 | microfibrillar-associated protein 3-like | MFAP3L |
| -1.57 | omega-3 fatty acid receptor 1 | O3FAR1 |
| -1.57 | somatostatin receptor 1 | SSTR1 |
| -1.56 | zinc finger protein 804B | ZNF804B |
| -1.55 | uncharacterized protein C11orf87 homolog | C11orf87 |
| -1.54 | Unidentified transcript |  |
| -1.52 | histone deacetylase 9 isoform 5 or 6 | HDAC9 |
| -1.51 | cadherin-7 | CDH7 |
| -1.51 | STAM binding protein-like 1 | STAMBPL1 |
| -1.51 | spermatogenesis associated, serine-rich 2-like | SPATS2L |
| -1.51 | F-box protein 43 | FBXO43 |
| -1.50 | ELAV (embryonic lethal, abnormal vision, Drosophila)-like 2 (Hu antigen B) | ELAVL2 |
| -1.50 | protein FAM19A1-like | FAM19A1-like |
